# An In-Run Automatic Demodulation Phase Error Compensation Method for MEMS Gyroscope in Full Temperature Range

**DOI:** 10.3390/mi15070825

**Published:** 2024-06-26

**Authors:** Jianpeng Wang, Gongliu Yang, Yi Zhou, Jiangyuan Zhang, Fumin Liu, Qingzhong Cai

**Affiliations:** 1 School of Instrumentation and Optoelectronic Engineering, Beihang University, Beijing 100191, China; qingzhong_cai@163.com; 2 Beijing Institute of Aerospace Control Device, Beijing 100854, China; fuminliu@sina.com; 3 Advanced Technology Research Institute, Zhejiang University, Hangzhou 310058, China; 4 School of Mechanical Engineering, Nanjing University of Science and Technology, Nanjing 210094, China; yizhou_mems@njust.edu.cn; 5 School of Instrument Science and Engineering, Southeast University, Nanjing 210018, China; zhjiangyuan@sina.com

**Keywords:** in-run, demodulation phase error, MEMS gyroscope, identification and compensation, bias instability, full temperature

## Abstract

The demodulation phase error will cause the quadrature error to be coupled to the rate output, resulting in performance deterioration of the MEMS gyroscope. To solve this problem, an in-run automatic demodulation phase error compensation method is proposed in this paper. This method applies square wave angular rate input to the gyroscope and automatically identifies the value of the demodulation phase error through the designed automatic identification algorithm. To realize in-run automatic compensation, the demodulation phase error corresponding to the temperature point is measured every 10 °C in the full-temperature environment (−40~60 °C). The relationship between temperature and demodulation phase error is fitted by a third-order polynomial. The temperature is obtained by the temperature sensor and encapsulated in the ceramic packages of the MEMS gyroscope, and the in-run automatic compensation is realized based on the fitting curve. The temperature hysteresis effect on the zero-rate output (ZRO) of the gyroscope is eliminated after compensation. The bias instability (BI) of the three gyroscopes at room temperature (25 °C) is reduced by four to eight times to 0.1°/h, while that at full-temperature environment (−40~60 °C) is reduced by three to four times to 0.1°/h after in-run compensation.

## 1. Introduction

MEMS gyroscopes are widely used inertial devices for measuring the rotational angular rate [1,2]. In comparison to conventional gyroscopes, the MEMS gyroscopes present several advantages, such as compact size, lightweight construction, low power consumption, cost-effectiveness, and scalability for mass production facilitated by microfabrication techniques [3,4,5]. Consequently, MEMS gyroscopes have been widely employed in diverse sectors, including autonomous vehicles, aerospace, defense, consumer electronics, and other fields [6,7,8]. The MEMS gyroscope is a mass-spring-damper oscillator that can be configured into two resonant modes: the drive mode and the sense mode. Typically, the drive mode of the gyroscope is maintained at resonance with a constant amplitude [9,10]. Rotating the gyroscope will generate Coriolis acceleration, which encodes rotational information [11]. The response of the gyroscope sense mode, driven by the Coriolis acceleration, enables the determination of angular rate measurements.

Despite all these advantages, the MEMS gyroscope is prone to fabrication errors stemming from micromachining technology [12,13]. In order to suppress the impact of these non-ideal properties, a control circuit incorporating error compensation mechanisms is essential. The non-ideal attributes of the MEMS gyroscope encompass frequency split, stiffness coupling, and damping coupling [14,15,16]. Frequency split has been identified as a factor that can significantly decrease the signal-to-noise ratio and mechanical sensitivity of the gyroscope. The frequency split can be effectively eliminated through the application of open-loop electrostatic frequency tuning. Stiffness coupling will produce quadrature error, and damping coupling will produce in-phase error [17,18]. These two kinds of errors will be separated by phase-sensitive demodulation.

Nevertheless, it is worth noting that, due to the change in ambient temperature, the frequency split of the gyroscope drifts and the associated demodulation phase changes to produce phase error [19,20,21]. Consequently, the quadrature error will be coupled to the rate output, resulting in the I/Q coupling of the demodulation and the drift of the zero-rate output (ZRO) [22,23]. Therefore, in order to improve the performance of the gyroscope, it is necessary to design an in-run automatic demodulation phase error compensation method to eliminate the I/Q coupling of the demodulation.

A method for compensating IQ coupling phase errors using a one-time frequency sweep was proposed in [24]. This approach involves measuring the phase error by identifying the phase corresponding to the peak amplitude of the drive mode during frequency sweeping and subsequently adjusting the reference phase of the phase-locked loop to compensate for the error. While experimental results demonstrate the effectiveness of this method in mitigating phase errors, it is noted that its applicability is limited as it cannot be implemented during the operation of the gyroscope. Furthermore, research on the influence of phase errors on quadrature cancellation techniques and zero-rate output in gyroscopes was proposed in [25]. The findings indicated that phase errors can significantly impact the ZRO of the gyroscope more than other system errors. In addition, another method for demodulating phase error compensation was introduced in [26]. This method utilizes a trained backpropagation (BP) neural network to predict phase errors based on resonant frequency and quadrature correction voltage. Experimental results illustrate the efficacy of this approach in addressing phase errors. However, its practicality is limited as it necessitates tailored training of the BP neural network and may not be suitable for most gyroscopes.

A method for calibrating I/Q phase errors in gyroscopes was introduced in a previous study [27]. This approach determines the phase error by evaluating the quadrature signal power against a predefined threshold and is specifically applicable to gyroscopes lacking quadrature suppression capabilities. Another technique involves self-correcting the phase based on the phase shift of the quadrature signal [28]. By leveraging the maximum quadrature error as an indicator of phase error, this method is adjusted using delay units to enhance the gyroscope’s temperature sensitivity in experimental settings. Additionally, a novel direct phase measurement and compensation method was proposed in [29], which operates by analyzing the phase variance between quadrature signals and the drive signal. This method intermittently suspends quadrature signal suppression at regular intervals to assess demodulation phase errors by comparing the phase disparity between the quadrature signal and the drive signal. However, this method requires continuous switching of the quadrature suppression loop, which cannot work when the quadrature suppression loop is turned off.

This paper analyzes the source of demodulation phase error and its influence on the gyro rate output. An in-run automatic demodulation phase error compensation method is proposed. The turntable is used to apply the square wave input angular rate to the gyro, and the demodulation phase error is automatically identified by the FFT and the designed algorithm. The advantages of this method are accurate identification and in-run compensation for phase error. The real-time temperature is obtained by a temperature sensor co-packaged with the MEMS gyroscope inside a ceramic shell, and the in-run compensation is realized by using the fitting curve obtained from the automatic test of multiple temperature points in the full temperature range.

Section 1 introduces the background of the MEMS gyroscope and previous research on demodulation phase error compensation. Section 2 introduces the operation principle of the MEMS gyroscope and the influence of the demodulation phase error. In Section 3, the method of in-run automatic phase error compensation in the full temperature range is proposed. Section 4 provides the experimental results of the proposed phase error compensation method and the performance before and after compensation.

## 2. Analysis of the Phase Error

### 2.1. Operation Principle of the MEMS Gyroscope

The MEMS gyroscope can be described by second-order differential equations [30].

(1) $$M\ddot{q}+D\dot{q}+Kq+2mA_{g}\Lambda \dot{q}=U\;M=\left[\begin{array}{cc} m & 0\\ 0 & m \end{array}\right],D=\left[\begin{array}{cc} d_{xx} & d_{xy}\\ d_{yx} & d_{yy} \end{array}\right],K=\left[\begin{array}{cc} k_{xx} & k_{xy}\\ k_{yx} & k_{yy} \end{array}\right],\Lambda =\left[\begin{array}{cc} 0 & -\Omega_{z}\\ \Omega_{z} & 0 \end{array}\right]$$

where $q={\left[\begin{array}{cc} x & y \end{array}\right]}^{T}$ is the displacement of the two modes of the gyroscope, $U={\left[\begin{array}{cc} F_{x} & F_{y} \end{array}\right]}^{T}$ is the electrostatic force applied to the two modes. In general, m is the proof mass of the gyroscope. $d_{xx}$ and $d_{yy}$ are the damping coefficients, $d_{xy}=d_{yx}$ are considered to be the damping coupling coefficients, $k_{xx}$ and $k_{yy}$ are the stiffness coefficients, $k_{xy}=k_{yx}$ are considered to be the stiffness coupling coefficients, $A_{g}$ is the angular gain that refers to the portion of proof mass that contributes to the generation of the Coriolis force, and $\Omega_{z}$ is the rotation rate in the Z axis.

For the convenience of calculation, both sides of Equation (1) are divided by mass *m* at the same time. Equation (1) can be rewritten as follows:

(2) $$\ddot{q}+d\dot{q}+kq+2A_{g}\Lambda \dot{q}=u\;d=\left[\begin{array}{cc} \frac{\omega_{x}}{Q_{x}} & \rho_{xy}\\ \rho_{yx} & \frac{\omega_{y}}{Q_{y}} \end{array}\right],k=\left[\begin{array}{cc} \omega_{x}^{2} & \omega_{xy}^{2}\\ \omega_{yx}^{2} & \omega_{y}^{2} \end{array}\right],\Lambda =\left[\begin{array}{cc} 0 & -\Omega_{z}\\ \Omega_{z} & 0 \end{array}\right],u=\left[\begin{array}{c} f_{x}\\ f_{y} \end{array}\right]$$

where $Q_{x}=m\omega_{x}/d_{xx}$, $Q_{y}=m\omega_{y}/d_{yy}$, $\omega_{x}=\sqrt{k_{xx}/m}$, $\omega_{y}=\sqrt{k_{yy}/m}$, $\omega_{xy}=\sqrt{k_{xy}/m}=\omega_{yx}$, and $\rho_{xy}=d_{xy}/m=\rho_{yx}$. During rate mode operation, the drive mode is activated and regulated by both the phase-locked loop (PLL) and automatic gain control (AGC) loop in order to achieve resonance at its natural frequency with a consistent amplitude. The drive voltage and the response of the drive mode can be expressed as follows:

(3) $$\begin{array}{c} V_{dx}=A_{dx}\cos\left(\omega_{x}t\right)\\ x(t)=A_{x}\sin(\omega_{x}t)\\ A_{x}=\frac{\eta_{vf}V_{dx}Q_{x}}{m\omega_{x}^{2}} \end{array}$$

where $\eta_{vf}$ is the voltage force conversion coefficient, $V_{dx}$ is the driving voltage of the drive mode, *x*(*t*) is the displacement of the drive mode, and $A_{x}$ is the amplitude of the displacement. The time domain response of the sense mode of the MEMS gyroscope can be described as follows:

(4) $$\begin{array}{c} y(t)=A_{I}\cos(\omega_{x}t+\varphi_{y})+A_{Q}\sin(\omega_{x}t+\varphi_{y})\\ A_{I}=A_{x}\omega_{x}\frac{2A_{g}\Omega_{z}+\rho_{xy}}{\sqrt{{\left(\omega_{y}^{2}-\omega_{x}^{2}\right)}^{2}+{\left(\omega_{y}\omega_{x}/Q_{y}\right)}^{2}}}\\ A_{Q}=A_{x}\frac{\omega_{xy}^{2}}{\sqrt{{\left(\omega_{y}^{2}-\omega_{x}^{2}\right)}^{2}+{\left(\omega_{y}\omega_{x}/Q_{y}\right)}^{2}}}\\ \varphi_{y}=-\tan^{-1}\frac{\omega_{y}\omega_{x}}{(\omega_{y}^{2}-\omega_{x}^{2})Q_{y}} \end{array}$$

The sense mode response *y*(*t*) can be decomposed into two components with 90° phases. By using two reference signals produced by a phase-locked loop (PLL), the response of the sense mode can be separated into two distinct outputs, denoted as *Q*(*t*) and *I*(*t*). One of these reference signals is synchronized in phase with the excitation mode displacement, whereas the other reference signal is in quadrature phase. The resulting demodulated outputs can be expressed as follows [18]:

(5) $$\begin{array}{l} Q(t)=A_{x}\frac{(2A_{g}\Omega_{z}+\rho_{xy})\omega_{x}\cos\varphi_{y}+\omega_{xy}^{2}\sin\varphi_{y}}{2\sqrt{{\left(\omega_{y}^{2}-\omega_{x}^{2}\right)}^{2}+{\left(\omega_{y}\omega_{x}/Q_{y}\right)}^{2}}}\\ I(t)=A_{x}\frac{-(2A_{g}\Omega_{z}+\rho_{xy})\omega_{x}\sin\varphi_{y}+\omega_{xy}^{2}\cos\varphi_{y}}{2\sqrt{{\left(\omega_{y}^{2}-\omega_{x}^{2}\right)}^{2}+{\left(\omega_{y}\omega_{x}/Q_{y}\right)}^{2}}} \end{array}$$

The equation above reveals that both demodulated outputs incorporate the non-ideal factors of the gyroscope and the rate information resulting from the phase shift induced by frequency division. The primary cause of performance degradation in the gyroscope is stiffness coupling. During rate mode operation, the demodulated output *Q*(*t*) is used to suppress the quadrature errors, while *I*(*t*) is employed for open-loop rate measurement. To suppress the coupling between the two demodulated outputs, it is imperative to ensure that the phase shift equals −90° by either eliminating the frequency split or adjusting the phase of the reference demodulation signal. In rate mode operation, the displacement of the quadrature error signal is suppressed to zero by the quadrature suppression loop. The stiffness coupling is eliminated by the DC suppression voltage, and the in-phase demodulation is used to measure the rotation rate. The motion equation of the sense mode can be described as follows:

(6) $$\ddot{y}+\frac{\omega_{y}}{Q_{y}}\dot{y}+\omega_{y}^{2}y+(\omega_{xy}^{2}-\mu V_{q}^{2})x+(2A_{g}\Omega_{z}+\rho_{xy})\dot{x}=0$$

where μ is the coefficient of the electrostatic negative stiffness effect. Solving the equation, it can be concluded that the suppression voltage $V_{q}$ is related to the stiffness coupling error. The suppression voltage, scale factor (SF), and zero rate output bias of the MEMS gyroscope are expressed as follows:

(7) $$V_{q}=\sqrt{\frac{\omega_{xy}^{2}}{\mu }},\;SF=A_{x}\frac{2A_{g}\omega_{x}}{2\sqrt{{\left(\omega_{y}^{2}-\omega_{x}^{2}\right)}^{2}+{\left(\omega_{y}\omega_{x}/Q_{y}\right)}^{2}}},\;Bias=\frac{\rho_{xy}}{2A_{g}}$$

In an ideal situation, the scale factor and quadrature suppression voltage are relatively independent based on Equation (7). Nonetheless, in practical applications, due to demodulation phase errors, these two variables become coupled rather than independent. Hence, the implementation of an in-run automatic compensation method is essential to effectively mitigate the impact of phase errors.

### 2.2. Analysis of the Phase Error

From Equation (4), the demodulation phase error is related to the resonate frequency, frequency split, and quality factor of the gyroscope. Equation (4) can be rewritten as follows:

(8) $$\varphi_{y}\approx -\tan^{-1}\frac{\omega_{x}}{2\Delta \omega Q_{y}}$$

**Figure 1:**
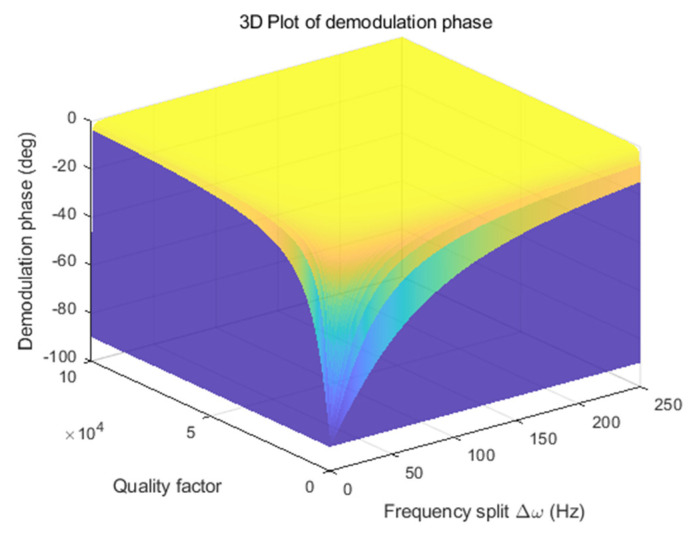
Relationship between demodulation phase, frequency split, and quality factor.

When the resonate frequency is 13,119 Hz, the demodulation phase at different quality factors and frequency splits is shown in Figure 1. It can be seen from the figure that with the increase in the quality factor, the demodulation phase change caused by frequency split becomes more intense, rapidly approaching zero degree. In addition, when the quality factor is constant, the increase in frequency split will also cause the demodulation phase to deviate from the ideal value by −90 degrees. For MEMS gyroscopes with lower quality factors, the demodulation phase error changes more slowly with increased frequency split. MEMS gyroscopes with low-quality factors are usually designed with a large frequency split for open-loop detection mode. The MEMS gyroscope reported in this paper is one of this type’s gyroscopes; thus, the resulting demodulation phase error of the MEMS gyroscope with low-quality factors usually cannot be neglected.

**Figure 2:**
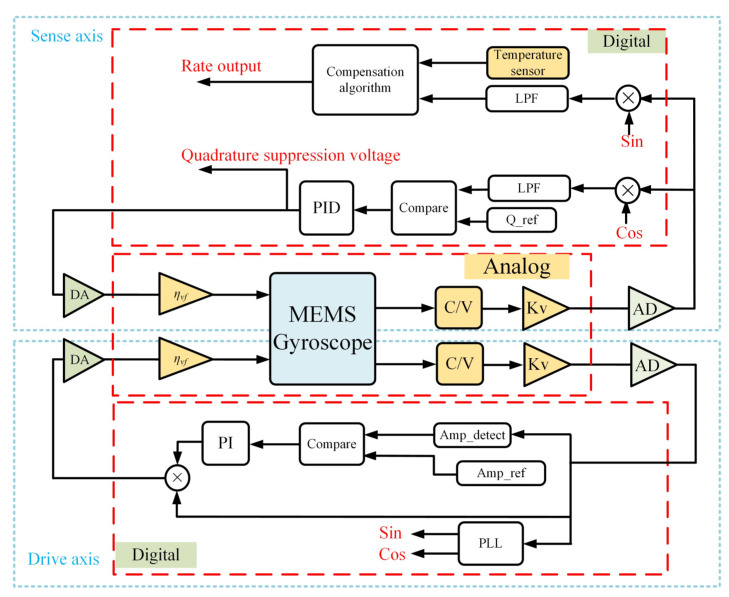
The entire control system of the MEMS gyroscope.

The entire control system of the MEMS gyroscope operating in rate mode is shown in Figure 2. The control system is composed of two closed loops. The self-excited closed loop is the basis of the control system, which is used to stabilize the resonance amplitude of the gyroscope drive mode. The quadrature suppression closed loop is used to suppress the stiffness coupling error. *Q*(*t*) is taken as the input of the quadrature suppression closed loop, and the PID controller generates the corresponding quadrature suppression voltage so that *Q*(*t*) is suppressed to zero. Considering the existence of quadrature suppression voltage and demodulation phase error, Equation (5) can be rewritten as follows:

(9) $$Q(t)=A_{x}\frac{(2A_{g}\Omega_{z}+\rho_{xy})\omega_{x}\cos\varphi_{y}+\left(\omega_{xy}^{2}-\mu V_{q}^{2}\right)\sin\varphi_{y}}{2\sqrt{{\left(\omega_{y}^{2}-\omega_{x}^{2}\right)}^{2}+{\left(\omega_{y}\omega_{x}/Q_{y}\right)}^{2}}}=0$$

After the quadrature suppression closed-loop is stable, that is, when *Q*(*t*) is suppressed to zero by the PID controller, Equation (9) can be solved as follows:

(10) $$V_{q}=\sqrt{\frac{(2A_{g}\Omega_{z}+\rho_{xy})\omega_{x}}{\mu \tan\left(\varphi_{y}\right)}+\frac{\omega_{xy}^{2}}{\mu }}$$

From the above equation, when the demodulation phase is misaligned, the quadrature suppression voltage is affected by the input angular rate and damping coupling error. According to the previous theoretical derivation and experimental results, the quadrature suppression voltage will inevitably affect the frequency split of the gyroscope, leading to the demodulation phase changing again and the degree of misalignment changing again. In addition, in this case, the demodulation output of the in-phase channel will also be affected. The *I*(*t*) in Equation (5) can be rewritten as follows:

(11) $$I(t)=A_{x}\frac{-(2A_{g}\Omega_{z}+\rho_{xy})\omega_{x}\sin\varphi_{y}+\left(\omega_{xy}^{2}-\mu V_{q}^{2}\right)\cos\varphi_{y}}{2\sqrt{{\left(\omega_{y}^{2}-\omega_{x}^{2}\right)}^{2}+{\left(\omega_{y}\omega_{x}/Q_{y}\right)}^{2}}}$$

After the quadrature suppression closed-loop is stable, Equation (11) can be solved as follows:

(12) $$I(t)=A_{x}\frac{-(2A_{g}\Omega_{z}+\rho_{xy})\omega_{x}}{2\sin\varphi_{y}\sqrt{{\left(\omega_{y}^{2}-\omega_{x}^{2}\right)}^{2}+{\left(\omega_{y}\omega_{x}/Q_{y}\right)}^{2}}}$$

Although the rate output is not directly affected by the stiffness coupling error, it will be indirectly affected by the stiffness coupling error through the demodulation phase. In this situation, the scale factor (SF) and zero rate output bias of the MEMS gyroscope are expressed as follows:

(13) $$\begin{array}{c} SF=A_{x}\frac{2A_{g}\omega_{x}}{2\sin\left(\varphi_{y}\right)\sqrt{{\left(\omega_{y}^{2}-\omega_{x}^{2}\right)}^{2}+{\left(\omega_{y}\omega_{x}/Q_{y}\right)}^{2}}}\\ ZRO=A_{x}\frac{\rho_{xy}\omega_{x}}{2\sin\left(\varphi_{y}\right)\sqrt{{\left(\omega_{y}^{2}-\omega_{x}^{2}\right)}^{2}+{\left(\omega_{y}\omega_{x}/Q_{y}\right)}^{2}}} \end{array}$$

From Equation (13), it can be observed that both the scale factor and the zero-rate output (ZRO) are amplified due to the demodulation phase error. However, the original ZRO and scale factor are affected by the changing demodulation phase. Although the normalized ZRO is unchanged, the scale factor is not measured in real time when the gyroscope is actually running, so the drift of ZRO is inevitable in the case of demodulation phase misalignment. Consequently, the gyroscope’s performance deteriorates. To address this issue, it is essential to design an automatic demodulation phase error compensation method for the MEMS gyroscope.

## 3. Design of the Demodulation Phase Compensation Method

### 3.1. Identification of the Demodulation Phase at Full Temperature

**Figure 3:**
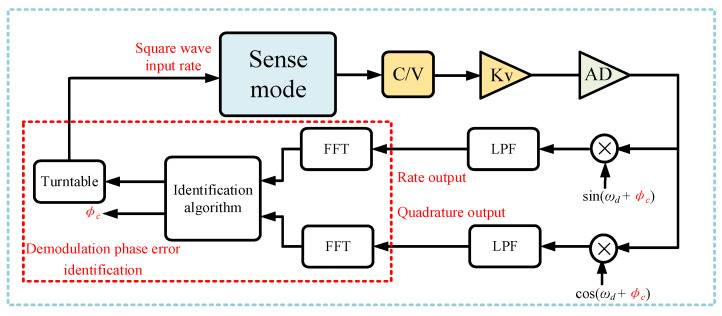
The system of automatic demodulation phase error identification.

According to Equation (5), when the demodulation phase is not aligned, the outputs of the in-phase channel and the quadrature channel are coupled with each other. In this case, if the gyroscope rotates at a square wave or sine wave-type angular rate, the output of the in-phase channel and the quadrature channel both have a square wave shape or a sine wave shape response. However, after demodulation phase error compensation, only the in-phase channel has a square wave shape or a sine wave shape response. Therefore, using this feature, an automatic demodulation phase error identification method is designed in this paper. The structure of the identification method is shown in Figure 3, which includes a turntable module, two fast Fourier transform (FFT) modules, and an automatic identification algorithm.

**Figure 4:**
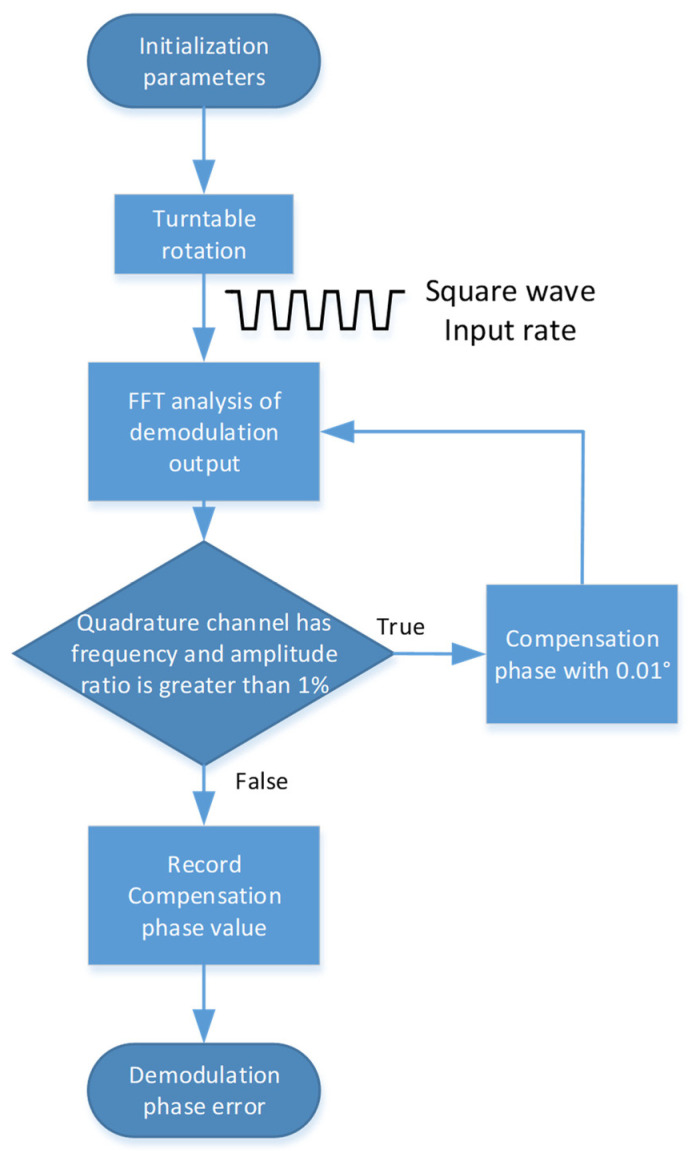
The automatic identification process of the proposed method.

The identification steps of the proposed automatic demodulation phase error identification method are shown in Figure 4. The following steps are involved in the compensation process:

1. The gyroscope and the control circuit are placed on the turntable, and the turntable can continuously output the angular rate of square wave shape through the designed automatic control program.
2. The demodulation signals of the quadrature channel and the in-phase channel are analyzed using the FFT module.
3. According to the FFT analysis results, determine whether there is an input frequency component in the quadrature channel and whether the amplitude ratio of the frequency component to the in-phase channel is greater than 1%. If there is, the compensation phase is increased by 0.01°, and the second step is followed again. If not, the compensated phase value is the identified demodulation phase error, and then the next step is continued.
4. The compensated phase value is recorded as the identified demodulation phase error.

In addition, the demodulation phase error of the MEMS gyroscope will change with the variation in temperature. In order to measure the demodulation phase error of the MEMS gyroscope at different temperatures, it is necessary to use the proposed identification method to identify the demodulation phase error at different temperature points in the −40~60 °C range. Three gyroscopes from the same batch were employed in the experiment, with their parameters at room temperature shown in Table 1. The gyroscopes have resonance frequencies around 13 kHz, quality factors around 3000, and a frequency split ranging from 233 to 299 Hz. According to Equation (8), the theoretical demodulation phase errors at room temperature can be calculated as 0.518°, 0.410°, and 0.462°, respectively.

**Figure 5:**
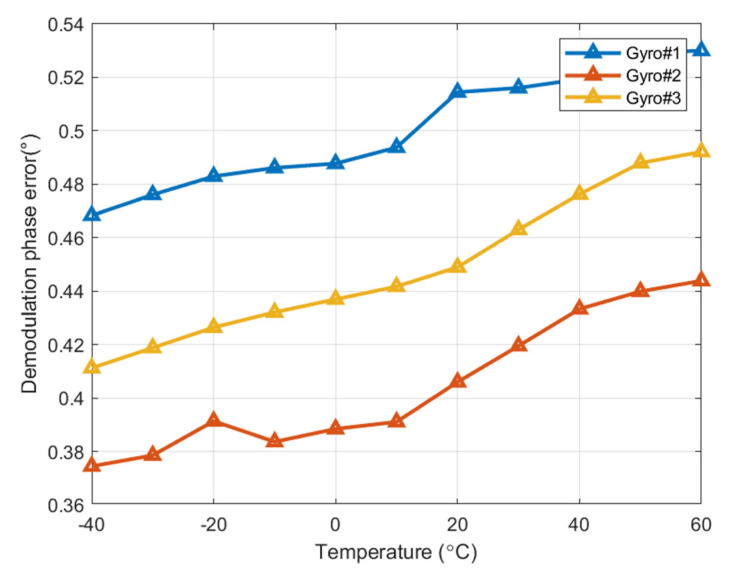
Experimental results of the demodulation phase error in the full temperature range of gyro#1, gyro#2, and gyro#3.

The MEMS gyroscope and control circuit are placed in the incubator. The temperature rise and fall rate of the incubator is set to 1 °C/min, and the temperature is kept for 30 min at each temperature point. The temperature is set from −40 °C to 60 °C, and demodulation phase error is collected every 10 °C. After 30 min of heat preservation at each temperature point, the demodulation phase error is identified and recorded by the proposed automatic demodulation phase error identification method. The experimental data are shown in Figure 5. The experimental results show that the demodulation phase error shows an approximate linear trend with temperature at full temperature, and the phase error value is small at low temperatures, while at high temperatures, the phase compensation value is large. At a temperature of 20 °C, the measured demodulation phase error closely matches the theoretical values in Table 1. This demonstrates that the phase error of the control circuit is small and can be neglected. The difference in demodulation phase error at the highest and lowest temperatures is 0.0617°, 0.0694°, and 0.0809°, respectively. The demodulation phase error at 20 °C has been used as the reference, and the maximum change rate is 12%, 16%, and 20%. Therefore, the demodulation phase error of the three gyroscopes varies greatly at full temperature range.

**Figure 6:**
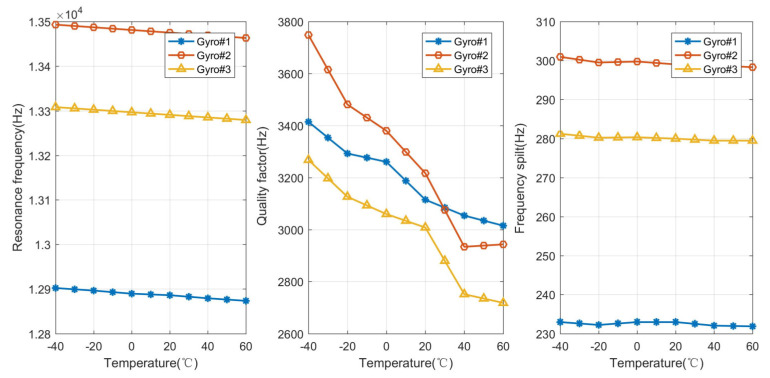
The resonant frequencies, quality factors, and frequency split of the three gyros were measured at different temperature points.

**Figure 7:**
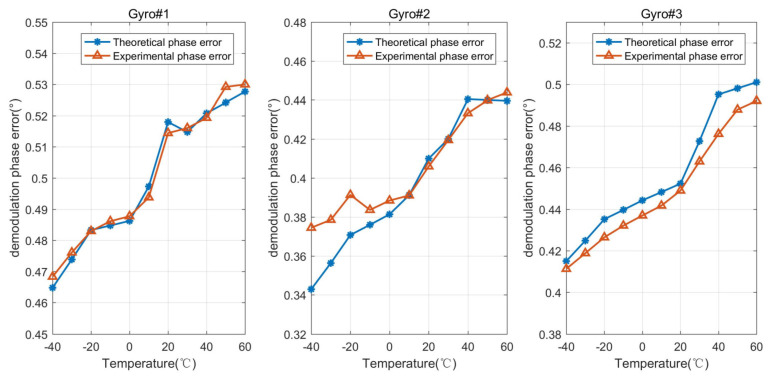
Theoretical phase errors and experimental results of the demodulation phase error in the full temperature range of gyro#1, gyro#2, and gyro#3.

The resonant frequencies, quality factors, and frequency split of the three gyros were measured at different temperature points. The measurement results are shown in Figure 6. According to the above measured parameters, the theoretical phase errors corresponding to different temperature points were calculated and compared with the experimental results of the tested three MEMS gyroscopes, as shown in Figure 7. As shown in Figure 7, the maximum error rate between the theoretical phase error and the actual measured phase error of Gyro#1 is 0.94% at 50 °C, and the minimum error rate is 0.065% at −20 °C. The maximum error rate between the theoretical phase error and the actual measured phase error of Gyro#2 is 8.43% at −40 °C, and the minimum error rate is 0.026% at 50 °C. The maximum error rate between the theoretical phase error and the actual measured phase error of Gyro#3 is 3.97% at 40 °C, and the minimum error rate is 0.74% at 20 °C. The comparison results prove that the main phase error is mechanical phase variation, and the phase error of the circuits can be ignored.

### 3.2. Compensation of the Demodulation Phase at Full Temperature

According to the previous experimental results and analysis, the demodulation phase error has an approximate linear relationship with temperature, but there are some nonlinear characteristics. Therefore, the relationship between demodulation phase error and temperature can be fitted by polynomial fitting.

For the varying demodulation phase error of the MEMS gyroscope in the full temperature range, the second- and third-order polynomial fitting methods are used to fit the demodulation phase error and the temperature of the MEMS gyroscope. The equation used for fitting is as follows:

(14) $$P=\rho_{3}T^{3}+\rho_{2}T^{2}+\rho_{1}T+\rho_{0}$$

**Figure 8:**
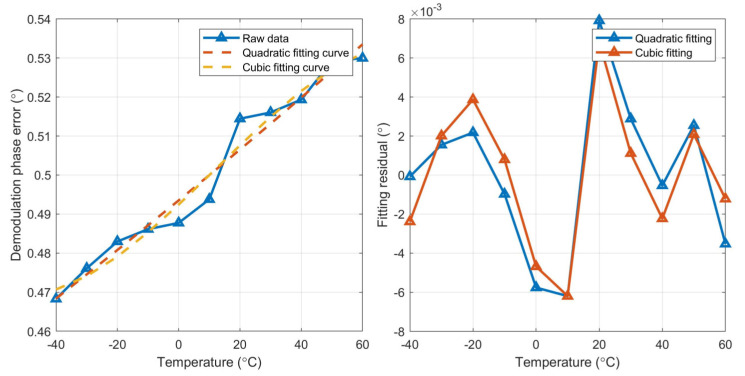
Fitting curve and fitting residual of gyro#1.

**Figure 9:**
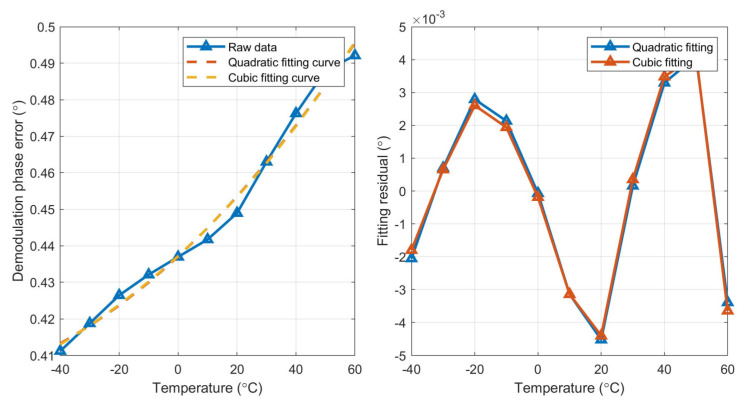
Fitting curve and fitting residual of gyro#2.

**Figure 10:**
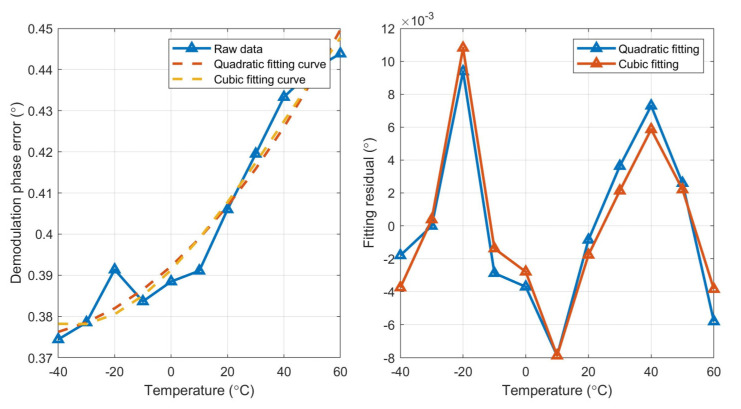
Fitting curve and fitting residual of gyro#3.

Second- and third-order polynomial fitting are performed on three MEMS gyroscopes, respectively, and the fitting curve and fitting residual of the three gyroscopes are shown in Figure 8, Figure 9 and Figure 10.

According to the fitting results, the fitting residual of the third-order (cubic) polynomial fitting is lower than that of the second-order (quadratic) fitting, so it is more suitable for the full temperature curve fitting of demodulation phase error. The curve obtained by fitting is saved on a ROM chip on the control circuit. The real-time temperature of the MEMS gyroscope is obtained through the designed temperature sensor inside the MEMS gyroscope. According to the obtained fitting curve and real-time temperature value, real-time demodulation phase compensation is carried out.

## 4. Experiment

**Figure 11:**
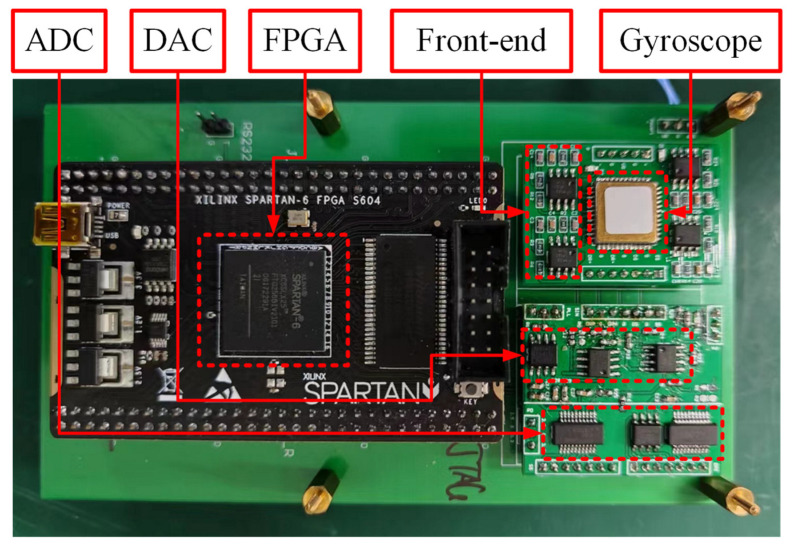
The control circuit board of the MEMS gyroscope.

The experimental circuit board depicted in Figure 11 accommodates the MEMS gyroscope, front-end circuit, analog-to-digital converter (ADC), digital-to-analog converter (DAC), digital isolator, flash chip, and FPGA chip, all seamlessly integrated on a printed circuit board (PCB). The front-end circuit, comprising an amplifier and a precise resistance, is designed as a transimpedance pre-amplifier circuit. To enhance sampling and control precision, a high-precision 16-bit ADC AD7902 with a sampling frequency of 500 kHz and a 16-bit DAC AD5541 with a clock frequency of 50 MHz are employed. A Spartan-6 XC65LX25 chip manufactured by Xilinx, San Jose, CA, USA was adopted as an FPGA chip. The control program is executed on the FPGA chip and stored on a flash chip. Given the system’s mixed-signal nature, digital noise is transferred to the analog domain, amplifying the analog noise levels. Using a digital isolator to segregate the digital and analog components effectively prevents digital noise interference. Data collected is transmitted to a computer via a communication port for subsequent storage and analysis, with a set sampling frequency of 1000 Hz. Furthermore, only +5 V power supplies are required and supplied through the communication port for the control circuit board.

**Figure 12:**
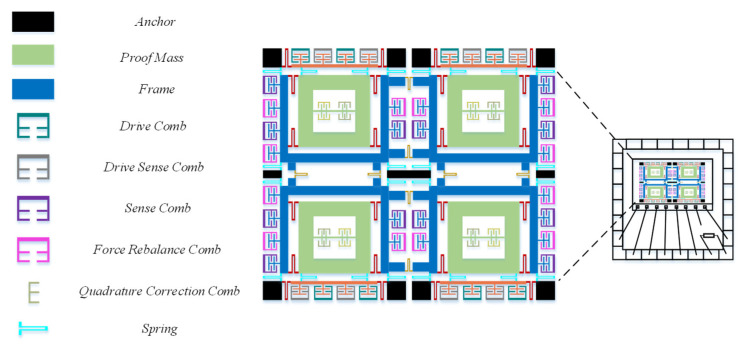
The structure of the MEMS gyroscope and the internal temperature sensor.

The temperature sensor and MEMS gyroscope structure are co-packaged within a ceramic shell, as illustrated in Figure 12. The temperature sensor used is the NCP15XH103D03RC NTC (negative temperature coefficient) thermistor manufactured by muRata Corporation, Tokyo, Japan with a resistance that varies with temperature. This temperature sensor, with dimensions of 1.00 mm × 0.50 mm, can be directly integrated into the ceramic tube shell of the MEMS gyroscope. It exhibits high measurement accuracy, with a resistance of 10 kΩ ± 0.5% at 25 °C, and is suitable for temperatures ranging from −40 °C to 125 °C. The measurement accuracy can reach up to 0.1 °C.

**Figure 13:**
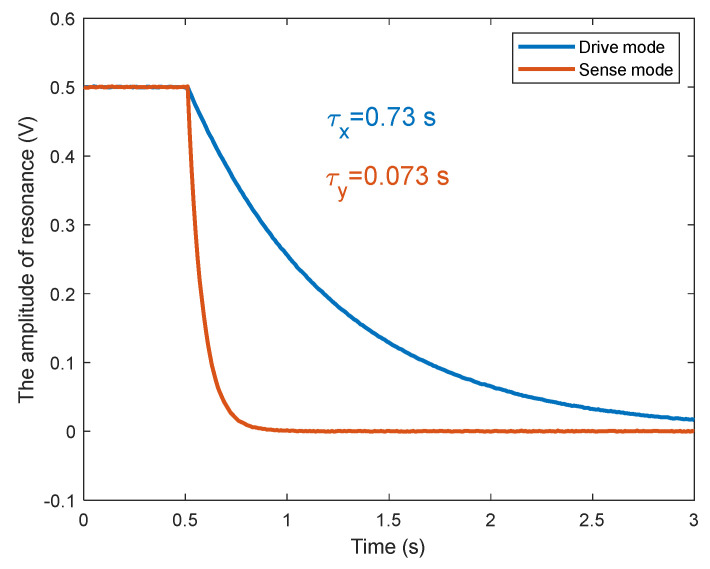
The ring-down experiment of the MEMS gyroscope.

The resonant frequency and decay time constant parameters of the MEMS gyroscope were measured by frequency sweep and ring-down experiments. The resonant frequency of the drive mode is 12,886 Hz ~ 13,291 Hz, the resonant frequency of the sensitive mode is 13,119 Hz ~13,571 Hz, and the initial frequency spike is 233 Hz ~ 299 Hz. The decay time constant of the gyroscope is measured by the ring down experiment, as shown in Figure 13. The quality factor of a gyroscope can be calculated using equation Q=πτf. The quality factor of gyroscope drive mode is around 30,000, and that of sense mode is around 3000.

**Figure 14:**
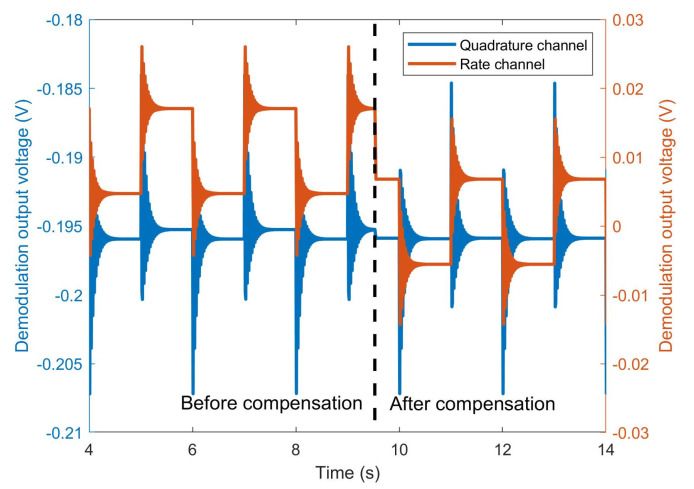
The output of the quadrature channel and rate channel of the MEMS gyroscope before and after compensation.

Using the designed automatic program to control the turntable to generate a square wave type rate, the input rate is set to 10°/s, the square wave period is 1.5 s, and the duty cycle is 50%. The demodulation output of the quadrature channel and rate channel are measured, as shown in Figure 14. Before compensation, there is a square wave-type response in the output of the quadrature channel. When phase error compensation is completed, although quadrature channel output is still affected by the transient input of the input rate, the output remains unchanged after stabilization and is no longer affected by the input angular rate. In addition, from Figure 14, it can be seen that there is an offset in the output of the rate channel before compensation, while the offset is eliminated to nearly zero after compensation. This is because the quadrature error is coupling into the rate channel before compensation, and the rate output is no longer affected by the quadrature error after compensation. Besides, the coupling damping error is much smaller than the quadrature error, so the offset after demodulation phase error compensation is approximately zero.

**Figure 15:**
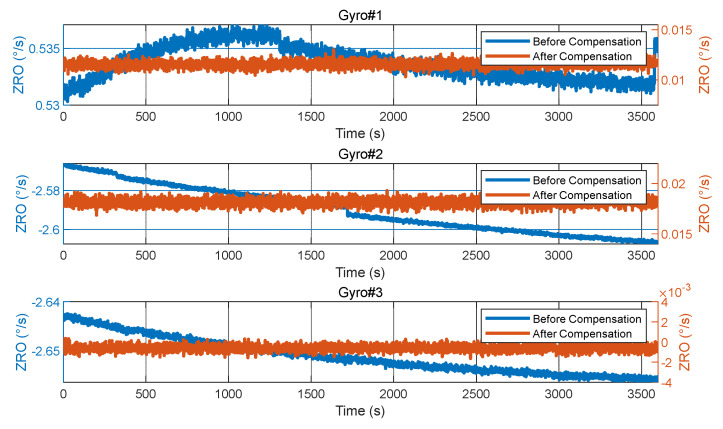
The comparison of ambient temperature ZRO before and after demodulation phase error compensation.

In order to verify the influence of demodulation phase error compensation on the zero-rate output (ZRO) performance of the three gyroscopes, the comparison of ZRO in the ambient temperature environment before and after compensation is shown in Figure 15. Before demodulation phase error compensation, the ZRO of the gyroscope is affected by the quadrature error. Because the quadrature error is larger than the coupling damping error, the bias of the ZRO before compensation is larger than that after compensation. In addition, the quadrature error is more sensitive to temperature changes than the coupling damping error, and the ZRO before compensation is more vulnerable to the influence of ambient temperature and drift than that after compensation.

**Figure 16:**
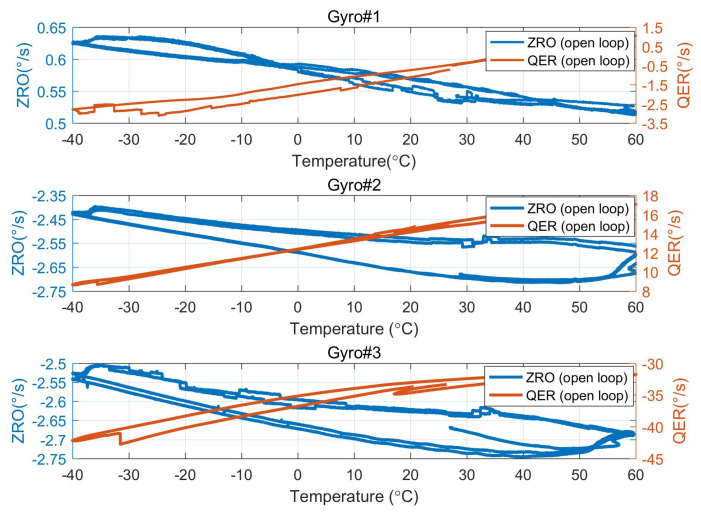
ZRO and quadrature equivalent rate (QER) over the full temperature range in open-loop detection mode.

In order to verify the influence of demodulation phase error compensation on the ZRO in the full temperature range of the gyroscope, the gyroscope and the control circuit are placed in an incubator, which is set to raise the temperature from −40 °C to 60 °C and then lower the temperature from 60 °C to −40 °C after holding 60 °C for 1 h. ZRO and quadrature equivalent rate (QER) over the full temperature range in open-loop detection mode are measured, as shown in Figure 16. As can be seen in Figure 16, the hysteresis of both QER and ZRO of the gyro exists, and the hysteresis of QER and ZRO is approximately in a mirror image relationship. The experimental results show that the ZRO hysteresis is somewhat affected by the quadrature error, but the quadrature error and the ZRO hysteresis curve are not completely symmetrical. This phenomenon indicates that the ZRO hysteresis is also affected by other factors, such as phase error.

**Figure 17:**
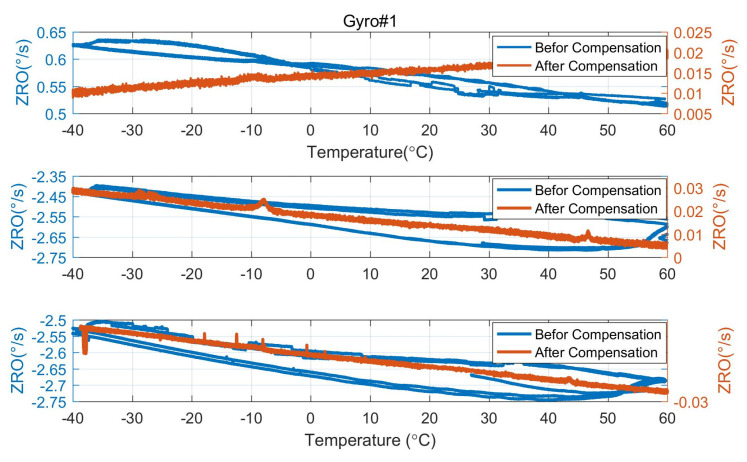
The comparison of ZRO in the full temperature range before and after demodulation phase error compensation.

The comparison of ZRO in the full temperature range before and after compensation is shown in Figure 17. As previously analyzed, the quadrature error of the gyroscope is more sensitive to temperature change than the coupling damping error. Therefore, in the experimental results, the variation of ZRO in the full temperature range of the three gyroscopes before demodulation phase error compensation is much greater than that after compensation. Besides, there is a temperature hysteresis phenomenon before compensation; that is, the variation of the ZRO during temperature rise is inconsistent with that during temperature drop. After the demodulation phase error compensation, the quadrature error no longer affects the rate output, and the coupling damping error is less sensitive to the temperature change. The residual hysteresis error of the ZRO is shown in Table 2. Hysteresis error is defined as the maximum difference between zero biases corresponding to the same temperature. The temperature at which the hysteresis error occurs is referred to as the temperature point in the table. The phase compensation reduced the temperature hysteresis of the three gyros by 95% to 98%.

**Figure 18:**
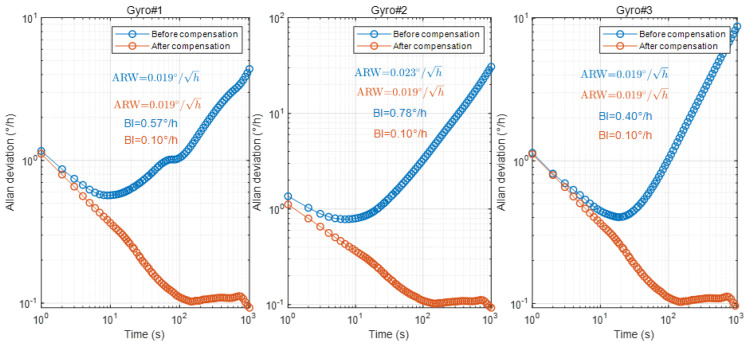
The Allan deviation analysis of the ZRO measured in an ambient temperature environment.

**Figure 19:**
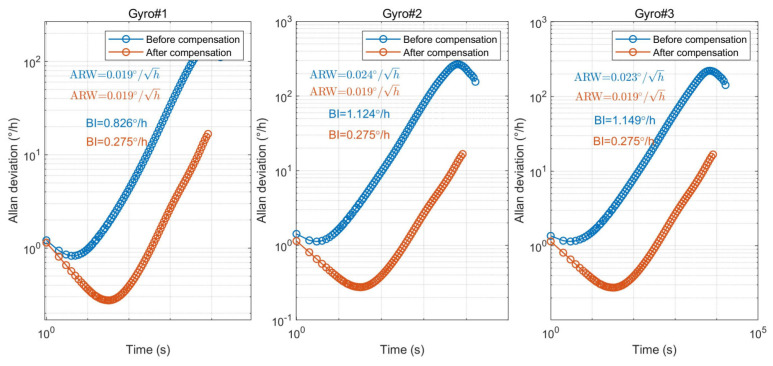
The Allan deviation analysis of the ZRO measured in a full-temperature environment.

In order to further evaluate the impact of the proposed demodulation phase error compensation method on the angle random walk (ARW) and the bias instability (BI) of the gyroscope, Allan variance analysis was performed on the ZRO measured in the ambient temperature environment (25 °C) and the ZRO measured in the full-temperature environment (−40~60 °C), as shown in Figure 18 and Figure 19. Allan variance analysis showed that the BI of the three gyroscopes decreased by 4~8 times to achieve 0.1°/h at room temperature after compensation. In a full-temperature environment, the BI of the gyroscope is decreased by three to four times to achieve 0.275°/h. The ARW of the three gyroscopes basically does not change in either an ambient or full-temperature environment because the demodulation phase error compensation has no effect on the noise floor and the noise source. According to the experimental results, the demodulation phase error compensation method proposed in this paper can effectively improve the BI performance of the MEMS gyroscope.

## 5. Conclusions

This paper reports an in-run automatic demodulation phase error compensation method that obtains the correlation curve by automatically identifying the error at different temperature points in the full temperature range, and third-order polynomial fitting is used to fit the curve. The real-time temperature is obtained by a temperature sensor co-packaged with the MEMS gyroscope inside a ceramic shell, and the in-run compensation is realized based on the fitting curve. The proposed method can effectively improve the BI of the MEMS gyroscope and eliminate the temperature hysteresis phenomenon in the process of temperature rise and fall. However, there are still some shortcomings. The proposed method can only be used in situations where an internal temperature sensor is present, whether it is inside the gyroscope or within the ceramic shell. The future research will focus on the in-run and automatic demodulation phase error compensation of MEMS gyroscopes without internal temperature sensors.

## Figures and Tables

**Table 1:** Parameters of the tested three MEMS gyroscopes.

	Gyro#1	Gyro#2	Gyro#3
*f_x_* (Hz)	12,886	13,475	13,291
*f_y_* (Hz)	13,119	13,774	13,571
Δ*f* (Hz)	233	299	280
*Q_y_*	3115	3217	3008
*φ_y_* (deg)	0.518	0.410	0.462

**Table 2:** The residual hysteresis of the zero bias.

	Maximum Temperature Point	Hysteresis	Maximum Temperature Point	Residual Hysteresis	Reduced Hysteresis
Gyro#1	29	0.028	−20	0.00139	95.04%
Gyro#2	40	0.178	−29	0.00354	98.01%
Gyro#3	32	0.121	48	0.00176	98.55%

## Data Availability

Data available on request due to restrictions.
